# Supplementary material for: Prevalence, pattern and risk factors for work-related musculoskeletal disorders among Nigerian plumbers
Source: PLoS One. 2022 Oct 26;17(10):e0273956. doi: 10.1371/journal.pone.0273956 (PMC9605337; doi:10.1371/journal.pone.0273956)
Supplement: S2 Questionnaire — (DOCX) [file pone.0273956.s002.docx]

**QUESTIONNAIRE**

# Project - Assessment of prevalence, pattern and risk factors for work-related musculoskeletal disorders among plumbers

We humbly solicit your participation in this research work by responding to the questions with utmost sincerity. All personal information will be treated with confidentiality and will be used strictly for the purpose of this research.

Thank you.

**Instructions:** Tick your answers from the options provided and fill in where necessary.

✔

**Section A: Socio-Demographic Data**

1. Age: ______________ years
2. Sex: Male Female
3. Marital status: Single Married Divorced Widow Widower
4. Highest educational level: Primary Secondary Tertiary
5. Years of experience: ______________ years
6. Working hours per day: _____________ hours
7. Job characteristics: **Tick all that applies**

Interprets blueprints and building specifications to map layout for pipes, drainage. ( )

Installs pipes and fixtures, such as sinks and toilets. ( )

Installs support for pipes, equipment and fixtures prior to installation. ( )

Assembles fittings and valves for installation. ( )

Modifies length of pipes, fixtures, and other plumbing materials as needed. ( )

Uses saws and pipe cutters as necessary. ( )

Installs heating and air conditioning systems including water heaters. ( )

Tests plumbing systems for leaks and other problems. ( )

Chooses plumbing materials based on budget, location and intended uses. ( )

Performs inspections and oversees other workers, such as apprentice. ( )

1. **Kindly tick job activities that are done often**

Interprets blueprints and building specifications to map layout for pipes, drainage. ( )

Installs pipes and fixtures, such as sinks and toilets. ( )

Installs support for pipes, equipment and fixtures prior to installation. ( )

Assembles fittings and valves for installation. ( )

Modifies length of pipes, fixtures, and other plumbing materials as needed. ( )

Uses saws and pipe cutters as necessary. ( )

Installs heating and air conditioning systems including water heaters. ( )

Tests plumbing systems for leaks and other problems. ( )

Chooses plumbing materials based on budget, location and intended uses. ( )

Performs inspections and oversees other workers, such as apprentice. ( )


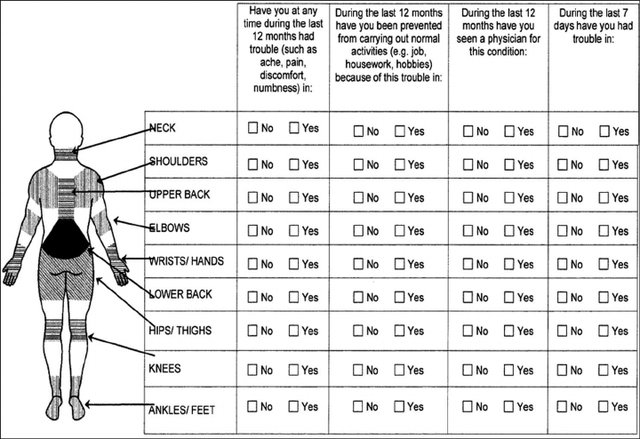


**SECTION B: Nordic Musculoskeletal Questionnaire**

**SECTION C: Job Factor Questionnaire**

This list describes things at work that could contribute to job-related pain and injury. Please indicate, on a scale of 0-10, how much of a problem each item (if any) is for you by ticking the appropriate number.

**0-1 = no problem** **2-7 = minor to moderate problem 8-10 = major problem**

| **SN** | **Item** | **0** | **1** | **2** | **3** | **4** | **5** | **6** | **7** | **8** | **9** | **10** |
| --- | --- | --- | --- | --- | --- | --- | --- | --- | --- | --- | --- | --- |
| 1. | Performing the same task over and over (hole drilling, cutting pipes, fitting hangers, digging/breaking of walls) |  |  |  |  |  |  |  |  |  |  |  |
| 2. | Working very fast for short periods (lifting, grasping, pulling, etc.) |  |  |  |  |  |  |  |  |  |  |  |
| 3. | Having to handle or grasp small objects |  |  |  |  |  |  |  |  |  |  |  |
| 4. | Insufficient breaks or pauses during the workday |  |  |  |  |  |  |  |  |  |  |  |
| 5. | Working in awkward or cramped positions |  |  |  |  |  |  |  |  |  |  |  |
| 6. | Working in the same position for long periods (standing, bending over, squatting, kneeling) |  |  |  |  |  |  |  |  |  |  |  |
| 7. | Bending or twisting your back in an awkward way |  |  |  |  |  |  |  |  |  |  |  |
| 8. | Working near or at your physical limits |  |  |  |  |  |  |  |  |  |  |  |
| 9. | Reaching or working over your head or away from your body |  |  |  |  |  |  |  |  |  |  |  |
| 10. | Hot, cold, humid, wet conditions |  |  |  |  |  |  |  |  |  |  |  |
| 11. | Continuing to work when injured or hurt |  |  |  |  |  |  |  |  |  |  |  |
| 12. | Carrying, lifting, or moving heavy materials or equipment |  |  |  |  |  |  |  |  |  |  |  |
| 13. | Work scheduling (overtime, irregular shifts, length of workday) |  |  |  |  |  |  |  |  |  |  |  |
| 14. | Using tools (design, weight, vibration, etc.) |  |  |  |  |  |  |  |  |  |  |  |
| 15. | Training on how to do the job |  |  |  |  |  |  |  |  |  |  |  |
