# Supplementary material for: Prevalence, pattern and risk factors for work-related musculoskeletal disorders among Nigerian plumbers
Source: PLoS One. 2022 Oct 26;17(10):e0273956. doi: 10.1371/journal.pone.0273956 (PMC9605337; doi:10.1371/journal.pone.0273956)
Supplement: Attachment — Submitted filename: PLOS ONE Review COMMENTS.docx.pdf [file pone.0273956.s003.pdf]

REVIEWER COMMENTS

This research primarily sought to assess the prevalence, pattern and associated risk factors for work-related musculoskeletal disorders among plumbers. In particular, authors reported key scientific results including a 12-month and 7-day prevalence of WMSDs as 84.6% and 50.8% respectively. The authors suggested the most affected body part to be in the order of; low-back>neck> knee.

The findings are original and have not been reported elsewhere.

The choice of assessment questionnaires and study design used was appropriate in addressing the aim of this research. The analysis and presentation of results are clear and addresses the research objectives.

Authors' conclusions clearly answered set objectives. However, it will be critical to recommend some practical, but cost-effective measures, given the high WMSD prevalences recorded among these plumbers, especially when most of them fall within the informal sector category.

The publication also followed the proper reporting requirements and community standards for data accessibility was written in clear, standard English, and complied with all applicable criteria for research ethics and integrity.

However the sentence "There is a moderate to good agreement for the job factor items and the stability of the instrument was evaluated to be 0.8 [28]" was miscited, given that paper referenced actually sought to "describe the identification and characterization of a novel human mitochondrial inner membrane protein homologous to the yeast Tim50", hence the deviation.
