# Supplementary material for: Prevalence, pattern and risk factors for work-related musculoskeletal disorders among Nigerian plumbers
Source: PLoS One. 2022 Oct 26;17(10):e0273956. doi: 10.1371/journal.pone.0273956 (PMC9605337; doi:10.1371/journal.pone.0273956)
Supplement: Attachment — Submitted filename: Response to reviewers - PLOSONE.docx [file pone.0273956.s004.docx]

PONE-D-22-22802

Prevalence, Pattern and Risk Factors for Work-Related Musculoskeletal Disorders among Nigerian Plumbers

Dear Editor,

Thank you for your careful consideration and review of our manuscript. Please find below the point-by-point responses to the comments.

| Editor | Comment | Response |
| --- | --- | --- |
|  | Please find more information on the policy and a link to download a blank copy of the questionnaire here: https://journals.plos.org/plosone/s/best-practices-in-research-reporting. Please upload a completed version of your questionnaire as Supporting Information when you resubmit your manuscript. | This has been done |
|  | We note that you have indicated that data from this study are available upon request. PLOS only allows data to be available upon request if there are legal or ethical restrictions on sharing data publicly. For more information on unacceptable data access restrictions, please see http://journals.plos.org/plosone/s/data-availability#loc-unacceptable-data-access-restrictions | Data will be made available. There is no legal restriction on sharing. |
|  | Please review your reference list to ensure that it is complete and correct. If you have cited papers that have been retracted, please include the rationale for doing so in the manuscript text, or remove these references and replace them with relevant current references. Any changes to the reference list should be mentioned in the rebuttal letter that accompanies your revised manuscript. If you need to cite a retracted article, indicate the article’s retracted status in the References list and also include a citation and full reference for the retraction notice. | This has been done. Corrected references are presented in red. Replaced references are in bold format (red). |
|  | The manuscript must describe a technically sound piece of scientific research with data that supports the conclusions. Experiments must have been conducted rigorously, with appropriate controls, replication, and sample sizes. The conclusions must be drawn appropriately based on the data presented. | This has been done. |
| Reviewer 1 |  |  |
|  | The authors need to do more rigorous work with this research. It is an interesting topic but the rationale and the justification do not sound strong. Research has been done on Musculoskeletal disorders and the link to Disability. How does it tie together? Why Plumbers? why not other artisans? It doesn't present a rich literature search proving that it is a study worth committing to. | This has been done (last paragraph of the introduction section – for the rationale) |
|  | The sample size is too small and cannot be not representative of the population of interest. Are there biases? How have they handled this? | Anecdotally, plumbers in Nigeria are relatively scarce. This information has been included in the manuscript. |
| Reviewer 2 |  |  |
|  | This is an interesting, well-written, well-organized and recommendable paper that examines the prevalence of musculoskeletal problems among plumbers in low- and middle-income countries. |  |
|  | This research primarily sought to assess the prevalence, pattern and associated risk factors for work-related musculoskeletal disorders among plumbers. In particular, authors reported key scientific results including a 12-month and 7-day prevalence of WMSDs as 84.6% and 50.8% respectively. The authors suggested the most affected body part to be in the order of; low-back>neck> knee. The findings are original and have not been reported elsewhere. The choice of assessment questionnaires and study design used was appropriate in addressing the aim of this research. The analysis and presentation of results are clear and addresses the research objectives. |  |
|  | Authors’ conclusions clearly answered set objectives. However, it will be critical to recommend some practical, but cost-effective measures, given the high WMSD prevalences recorded among these plumbers, especially when most of them fall within the informal sector category. The publication also followed the proper reporting requirements and community standards for data accessibility was written in clear, standard English, and complied with all applicable criteria for research ethics and integrity. However the sentence “There is a moderate to good agreement for the job factor items and the stability of the instrument was evaluated to be 0.8 [28]” was miscited, given that paper referenced actually sought to “describe the identification and characterization of a novel human mitochondrial inner membrane protein homologous to the yeast Tim50”, hence the deviation. | The suggested recommendation has been included in the discussion section.  The reference [28] has been confirmed. It is presently reference [37] in this revision. |
| Editor |  |  |
|  | While revising your submission, please upload your figure files to the Preflight Analysis and Conversion Engine (PACE) digital diagnostic tool, <https://pacev2.apexcovantage.com/>. PACE helps ensure that figures meet PLOS requirements. To use PACE, you must first register as a user. Registration is free. Then, login and navigate to the UPLOAD tab, where you will find detailed instructions on how to use the tool. If you encounter any issues or have any questions when using PACE, please email PLOS at [figures@plos.org](mailto:figures@plos.org). Please note that Supporting Information files do not need this step. |  |

All new inclusion are presented in bold format. References were reformatted to suit the journal style. The questionnaires and data have been uploaded as appendix.

Thank you.

Chidozie Mbada
